# Reduced levels of protein recoding by A-to-I RNA editing in Alzheimer's disease

**DOI:** 10.1261/rna.054627.115

**Published:** 2016-02

**Authors:** Khen Khermesh, Anna Maria D'Erchia, Michal Barak, Anita Annese, Chaim Wachtel, Erez Y. Levanon, Ernesto Picardi, Eli Eisenberg

**Affiliations:** 1 Mina and Everard Goodman Faculty of Life Sciences, Bar-Ilan University, Ramat-Gan 59002, Israel; 2 Department of Biosciences, Biotechnology and Biopharmaceutics, University of Bari, Bari, 70126, Italy; 3 Institute of Biomembranes and Bioenergetics, National Research Council, Bari, 70126, Italy; 4 Sagol School of Neuroscience and Raymond and Beverly Sackler School of Physics and Astronomy, Tel Aviv University, Tel Aviv, 69978, Israel

**Keywords:** Alzheimer disease, epigenetics, RNA editing, targeted resequencing

## Abstract

Adenosine to inosine (A-to-I) RNA editing, catalyzed by the ADAR enzyme family, acts on dsRNA structures within pre-mRNA molecules. Editing of the coding part of the mRNA may lead to recoding, amino acid substitution in the resulting protein, possibly modifying its biochemical and biophysical properties. Altered RNA editing patterns have been observed in various neurological pathologies. Here, we present a comprehensive study of recoding by RNA editing in Alzheimer's disease (AD), the most common cause of irreversible dementia. We have used a targeted resequencing approach supplemented by a microfluidic-based high-throughput PCR coupled with next-generation sequencing to accurately quantify A-to-I RNA editing levels in a preselected set of target sites, mostly located within the coding sequence of synaptic genes. Overall, editing levels decreased in AD patients’ brain tissues, mainly in the hippocampus and to a lesser degree in the temporal and frontal lobes. Differential RNA editing levels were observed in 35 target sites within 22 genes. These results may shed light on a possible association between the neurodegenerative processes typical for AD and deficient RNA editing.

## INTRODUCTION

It has been increasingly appreciated in recent years that epigenetic transcriptome diversity plays a major role in complex organisms. Alternative splicing, occurring in the vast majority of mammalian primary transcripts, is considered to be a key contributor to that diversity (Johnson et al. 2003; Sultan et al. 2008). An additional co/post-transcriptional process that appears to be widespread in mammals is RNA editing.

Of the various types of RNA editing, A-to-I base modification is the most common in higher eukaryotes (Bass 2002; Nishikura 2010; Savva et al. 2012; Li and Church 2013). It enzymatically deaminates individual adenosine (A) bases in the pre-mRNA to a modified base, inosine (I), which is recognized as guanosine (G) by the cell translational apparatus. A-to-I RNA editing is catalyzed by the ADAR (adenosine deaminase acting on RNA) family of enzymes, acting mostly in a site-specific manner on dsRNA structures formed in the pre-mRNA molecules (Patterson and Samuel 1995; Melcher et al. 1996; Lehmann and Bass 2000). Three ADAR gene family members (ADAR1–3) have been identified in mammals. ADAR (ADAR1) and ADARB1 (ADAR2) can be detected in many tissues, while ADARB2 (ADAR3) seems to be restricted to the brain (Jacobs et al. 2009; Horsch et al. 2011; Savva et al. 2012). It is believed that ADAR and ADARB1 are the main catalytic enzymes that are accountable for all A-to-I editing activity, while the third member, ADARB2 (ADAR3), lacks a catalytic domain and its function is yet unclear (Chen et al. 2000). ADAR has two distinct splice variants: the ADAR-p150 variant, transcribed from an IFN-inducible promoter and detected mainly in the cytoplasm, and the constitutively expressed ADAR-p110 variant that is localized exclusively in the nucleus (Patterson and Samuel 1995).

Millions of genomic sites are targeted by ADARs in humans, but the vast majority resides in *Alu* repetitive elements within untranslated regions (Athanasiadis et al. 2004; Blow et al. 2004; Kim et al. 2004; Levanon et al. 2004; Barak et al. 2009; Bazak et al. 2014a). Many possible biological roles for A-to-I RNA editing in noncoding sequences has been discussed in the literature (Prasanth et al. 2005; Scadden 2005, 2007; Hundley et al. 2008; Chen and Carmichael 2009). However, naturally, much interest has been focused on the minute fraction of editing sites in protein coding genes that result in amino acid substitutions, also known as the recoding sites (Sommer et al. 1991; Niswender et al. 1999; Berg et al. 2001; Hoopengardner et al. 2003; Iwamoto and Kato 2003; Bhalla et al. 2004; Cenci et al. 2008; Schellekens et al. 2012). Recoding by A-to-I editing is a highly regulated process, whereas deregulated recoding has been associated with multiple ailments (Paz et al. 2007; Slotkin and Nishikura 2013; Tomaselli et al. 2014; Paz-Yaacov et al. 2015). Only a few dozen recoding sites are conserved across mammals, and hundreds of human-specific (or primate-specific) sites have been identified, mostly weakly edited (Li et al. 2013; Pinto et al. 2014; Ramaswami and Li 2014).

Notably, many of the well-characterized recoding sites reside in brain-specific transcripts associated with neuronal functions. Several investigations have associated altered recoding levels in specific sites with various neurological and neurodegenerative disorders (Niswender et al. 2001; Vissel et al. 2001; Maas et al. 2006; Tariq and Jantsch 2012; Slotkin and Nishikura 2013; Gaisler-Salomon et al. 2014; Li et al. 2014), such as major depression, epilepsy, schizophrenia, amyotrophic lateral sclerosis, and Alzheimer's disease (Feldmeyer et al. 1999; Kortenbruck et al. 2001; Vollmar et al. 2004; Iwamoto et al. 2005, 2009). However, so far, only a small fraction of recoding sites has been characterized and investigated in a pathological context. Many studies have focused on the editing alteration of Q/R site in GRIA2 gene of AMPA receptor, but for most sites close to nothing is known on the effect of recoding on the resulting proteins and the downstream effect on cell function (Kawahara et al. 2006; Kwak et al. 2008). Given the association between recoding and the nervous system, it is natural to explore altered recoding activity in specific brain pathologies across multiple recoding sites.

Alzheimer disease ([AD] OMIM #104300) accounts for over 50% of all dementia cases, presently affecting more than 24 million people worldwide (Alzheimer Association 2014). It is characterized by a progressive decline in cognitive function, which typically begins with deterioration in memory. The neuropathological hallmarks of the AD brain comprise extracellular precipitations of the β-amyloid peptide, which is derived from the amyloid precursor protein (APP) by proteolytic cleavage, and the presence of neurofibrillary inclusions composed of an abnormally phosphorylated and aggregated microtubule-associated τ protein. As a consequence of neurofibrillary inclusions, AD is accompanied by the progressive loss of neurons and inflammation around the senile plaques with reactive accumulation of microglial cells. Nevertheless, the etiological mechanisms underlying the neuropathological changes in AD remain unclear (Serrano-Pozo et al. 2011). Neuroprotective strategies should be implemented prior to neuronal loss and degeneration.

However, this requires preclinical markers to identify susceptible patients and early pathogenic mechanisms to serve as therapeutic targets. Transcriptomic analyses, that assume no a priori etiological hypotheses, promise much in elucidating the pathogenesis of complex diseases like AD and providing novel bio-markers.

Here we study globally the recoding activity in AD patients, looking for aberrations in the corresponding editing profile. We have selected 118 RNA editing sites located in 72 genes, most of which are located in coding regions and lead to amino acid substitution. The selected target-set is enriched in sites that are conserved through mammalian evolution. We then utilized an advanced targeted resequencing system that is built around the Fluidigm Access-Array (Fl-AA) platform, for microfluidic-based amplification of selected target regions across a multi samples panel (mmPCR) followed by in-parallel next-generation sequencing, to compare the editing profile between normal and AD brain tissues (Zhang et al. 2014).

## RESULTS

### Compiling the A-to-I editing sites target-set that is used in the targeted resequencing protocol

In the first two decades of RNA editing study, the method of choice for editing detection was low throughput PCR-based saturated amplification of a single locus, followed by Sanger DNA sequencing. Analyzing sequencing chromatograms, one may detect and quantify editing levels in the given site, with a typical accuracy of ∼5% (Li et al. 2009; Picardi et al. 2012). Introduction of RNA-seq techniques has led to massive enrichment in newly discovered A-to-I editing sites (Bahn et al. 2012; Park et al. 2012; Peng et al. 2012; Ramaswami et al. 2012; Bazak et al. 2014a,b). However, RNA-seq is generally less accurate in quantifying RNA editing levels due to the moderate read coverage currently available (Ramaswami et al. 2013). Several attempts have been made to couple targeted amplification and next-generation sequencing for accurate quantification of RNA editing with limited success (Li et al. 2009; Sanjana et al. 2012; Ramaswami et al. 2013). Very recently, a more robust experimental system which combines microfluidics-based multiplex PCR has been presented by Zhang et al. (2014). This innovative method comes with several benefits over previous approaches. Microfluidics enables the parallel analysis of multiple samples and target sites, while targeted deep sequencing provides sufficient per-base depth to allow reliable quantification for all sites studied.

Here, we apply for the first time this targeted resequencing strategy in order to investigate potential RNA editing alterations in specific brain regions obtained from AD brains. Since this methodology is PCR based, the first step of the experimental procedure was to carefully select a relevant subset of RNA editing targets. We searched the RADAR database (v.2), which stands as a comprehensive collection of rigorously annotated RNA editing sites, for editing sites residing out of *Alu* repeats (Ramaswami and Li 2014). The selected sites were then filtered according to the following five guiding principles: preference for sites (i) located within protein coding regions, (ii) resulting in nonsynonymous amino acid changes, (iii) exhibiting evolutionarily conserved editing in at least one of the three mammals: chimpanzee, rhesus, and mouse, (iv) located in genes associated with neuronal functions and plasticity, and (v) sites that previously had recorded levels of RNA editing. The final list of targeted sites included 118 sites that are located in 72 different genes (Supplemental Table S1). Of these, 98 sites are located in protein coding regions of 67 genes, nine sites are in the 3′-UTR of two genes and 11 sites reside in three noncoding RNA genes (lncRNAs). Overall, the selected target-set represented a comprehensive panel of RNA editing sites well-positioned to assess A-to-I editing recoding capacity.

Notably, our target-set included RNA editing positions located in membrane receptors and ion channels known to play relevant biological roles in maintaining the correct neuronal physiology and cellular homeostasis.

### Comprehensive and site-specific differential RNA editing levels detected in AD and NDC

The microfluidics-based multiplex PCR (mmPCR) approach for targeted resequencing is built around the Fluidigm Access Array, which enables uniform amplification of 48 different PCR products from each of the 48 RNA samples in a single experiment (Zhang et al. 2014). One may put more than one primer set in each well, increasing the number of amplicons per experiment even further. Output PCR amplicons are labeled with barcode sequences specific for each sample by a subsequent PCR and finally pooled for deep sequencing in order to obtain high coverage that allows for an accurate measurement of A-to-I RNA editing levels per genomic locus. Here, we used this platform to measure editing levels of the selected target-set of human editing sites in multiple diseased (AD) and in age- and sex-matched nondemented healthy controls (NDC) samples, originating from three brain regions: hippocampus (HpC), temporal lobe (TL) and frontal lobe (FL). The specific brain regions were chosen because they are known to be affected along the progression of the disease (Ray and Zhang 2010; Serrano-Pozo et al. 2011). Indeed, HpC is one of the primary and major brain locations damaged by extracellular precipitations of the β-amyloid peptide, showing extended neurodegeneration. Gradually, pathological lesions advance devastating temporal and frontal areas of neocortex.

We assayed all 118 editing sites that were primarily targeted. Of these, we successfully amplified and identified by sequence alignment 115 targeted sites (113 in HpC samples, 108 in TL and 107 in FL; Supplemental Table S2). To allow for accurate quantification of editing levels, we discarded all measurements with read coverage <500 reads, and considered only sites for which at least five normal and five diseased samples (of a given brain tissue-type) passed this cutoff, resulting in 66 target sites that were further analyzed (Supplemental Table S2).

**FIGURE 1.**
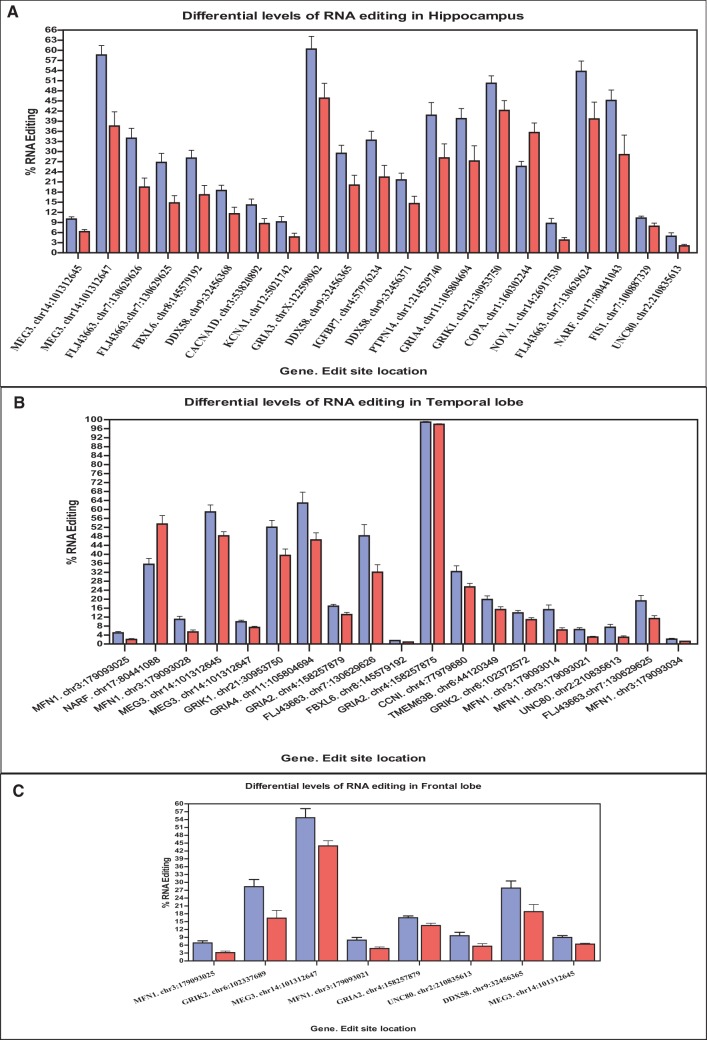
RNA editing target sites that show significant differential levels of NDC and AD. (*A*) In Hippocampus, 21 editing target sites exhibiting differential levels of A-to-I RNA editing were detected in AD (red bars) and in NDC (blue bars). Twenty sites show hypo-editing in AD and one site (COPA. Chr1:160302244) shows hyper-editing (*n* = 28 for AD, *n* = 20 in NDC). (*B*) In temporal lobe, 19 editing target sites exhibiting differential levels of A-to-I RNA editing were detected in AD (red bars) and NDC (blue bars). Eighteen sites show hypo-editing in AD and one site (NARF. Chr17:80441088) showing hyper-editing (*n* = 13 for AD, *n* = 11 in NDC). (*C*) In frontal lobe, eight editing sites detected in AD (red bars) and NDC (blue bars). All sites show hypo-editing in AD (*n* = 13 for AD, *n* = 11 in NDC). All values represented as mean + SEM.

In HpC, we found 21 differentially edited sites (Table 1; Fig. 1A), 20 of which are under edited in AD compared with NDC. The global editing signal, as measured by the mean editing level across all 66 sites was also reduced in AD (21.8% ± 4.5% in AD, 26.8% ± 3.5% in NDC, *P* = 0.03, *t*-test). This result is at odds with the fact that the expression levels of all three ADAR variants (ADAR-p110, ADAR-p150 and ADARB1) are at least as high in AD as they are in NDC (Fig. 4). Similar results were found for TL and FL. In TL, 19 target sites showed significant differential editing levels (Table 2; Fig. 1B), 18 of which exhibiting under-editing in AD. In FL, only eight sites show statistically significant differential editing levels in AD compare with NDC (Table 3; Fig. 1C), with all sites exhibiting hypo-editing in AD. In addition, the global editing signal was reduced in AD in both brain regions: 22.5% ± 4.8% in AD versus 24.4% ± 4% in NDC for TL (*P* = 0.002; *t*-test), and 23.5% ± 4% in AD versus 27% ± 2.6% in healthy controls (NDC) in FL (*P* = 7.0 × 10^−8^; *t*-test). Interestingly, three hypo-edited sites (in the UNC80 and MEG3 genes) were consistently detected in all three tissues (Table 6).

**TABLE 1.**
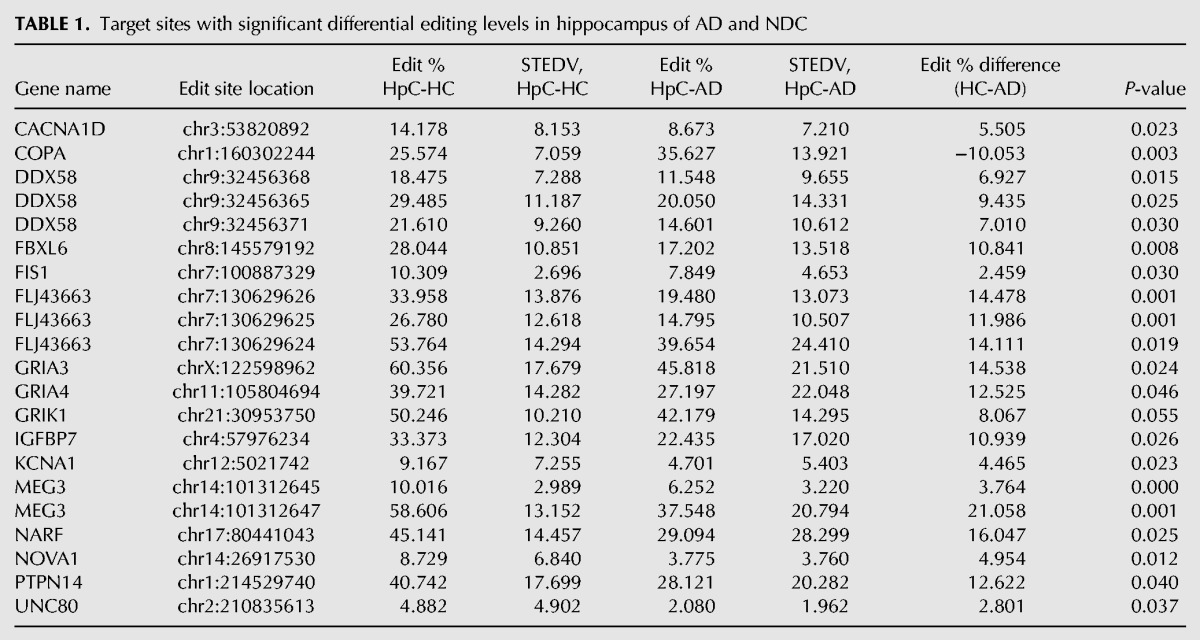
Target sites with significant differential editing levels in hippocampus of AD and NDC

**TABLE 2.**
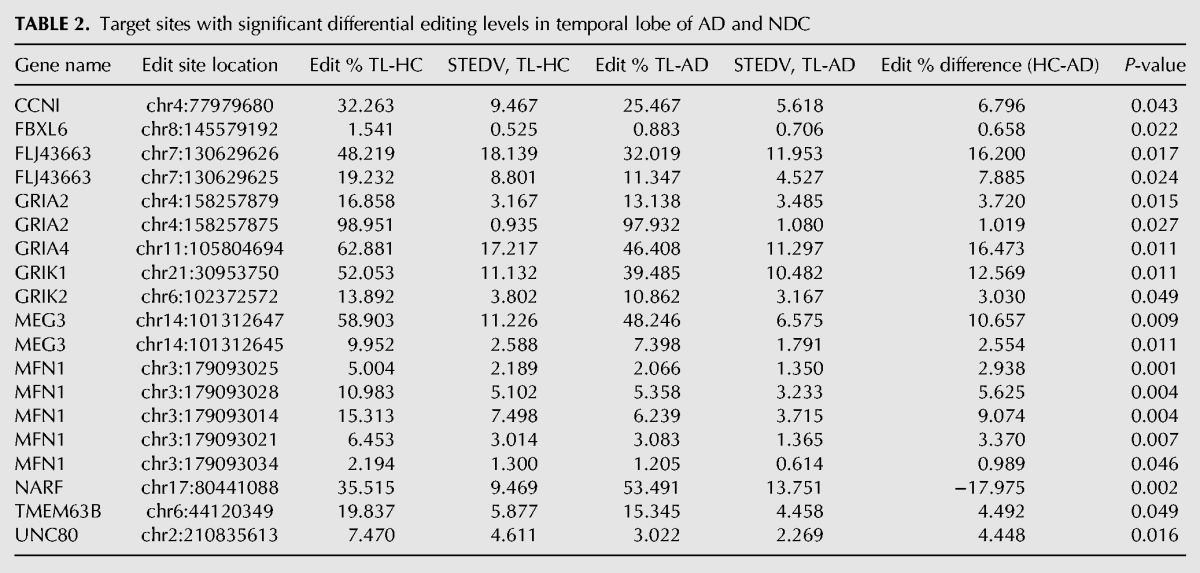
Target sites with significant differential editing levels in temporal lobe of AD and NDC

**TABLE 3.**
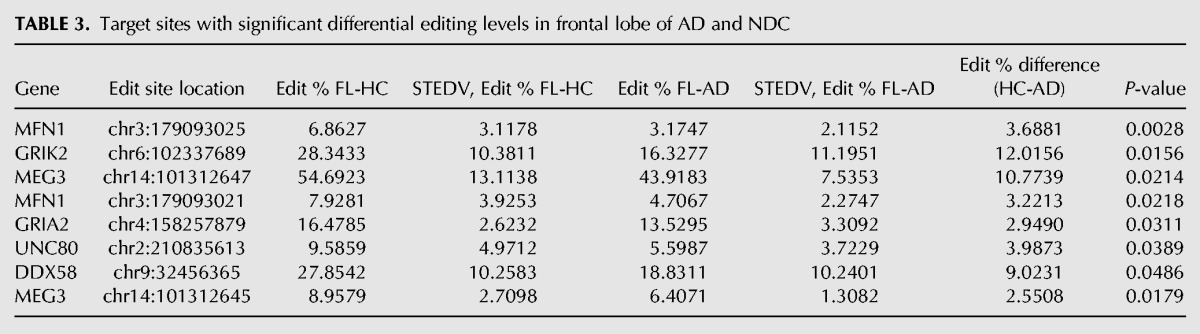
Target sites with significant differential editing levels in frontal lobe of AD and NDC

Our target-set of RNA editing sites includes 32 positions for which the editing level is reported in the RADAR database (Ramaswami and Li 2014). We have verified (Supplemental Table S4) that the editing levels recorded for this group of sites correlate with those measured in our study (bearing in mind differences in the brain region of origin and their characteristics).

### Analysis of clustered RNA editing sites

**FIGURE 2.**
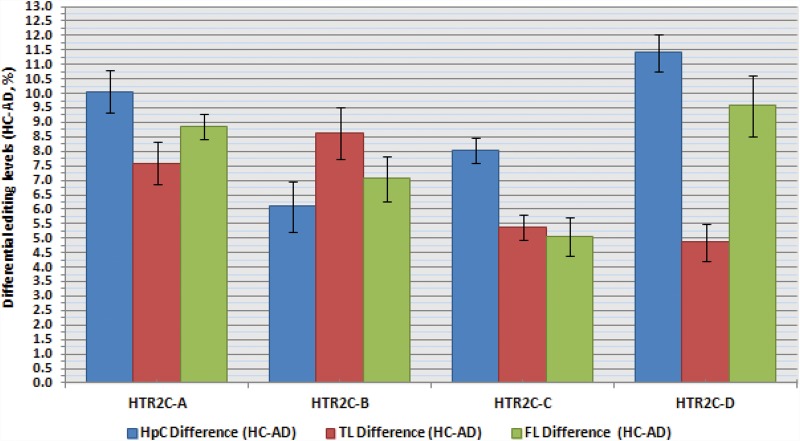
Differential levels of RNA editing between AD and NDC for the predominant sites of HTR2C. The results show hypo-editing of the four predominant sites of HTR2C (sites *A*–*D*) in AD compared with NDC in hippocampus (blue bars), temporal lobe (red bars), and frontal lobe (green bars). The graph points to a trend in the recorded editing levels, although none of these results were found to be statistically significant. Values are represented as mean ± SEM.

**FIGURE 3.**
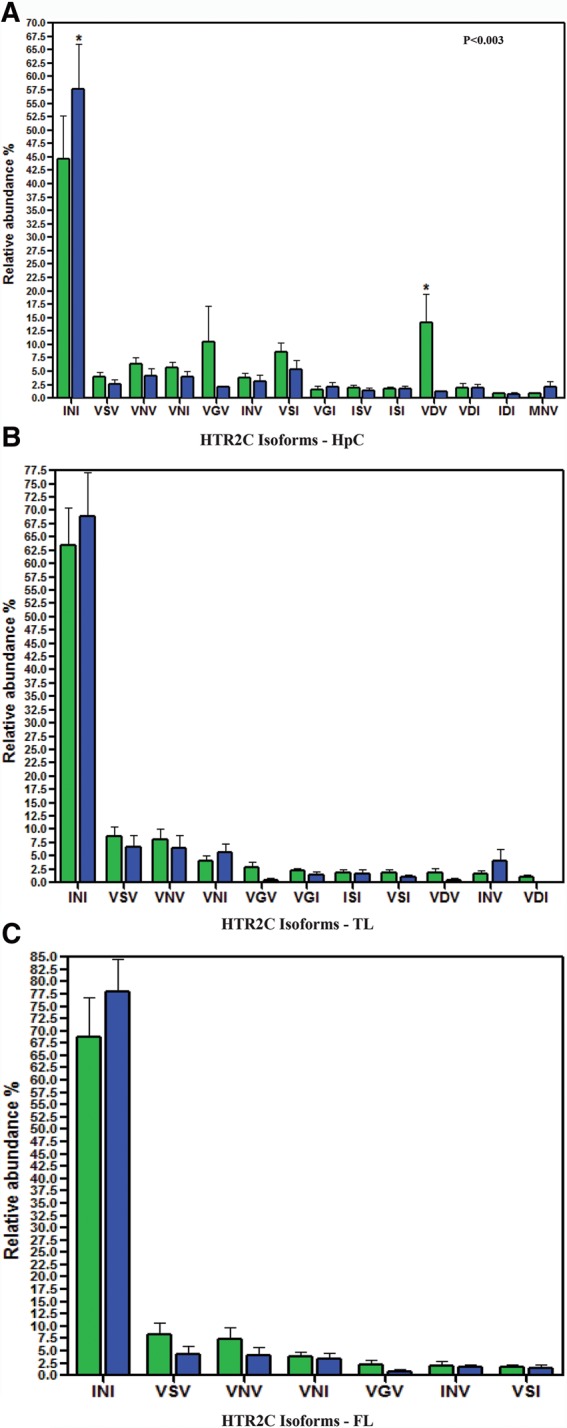
Relative abundance of HTR2C mRNA variants formed by RNA editing. The relative abundance of HTR2C mRNA variants formed by RNA editing detected in each brain region in AD (green bars) and NDC (blue bars). (*A*) Hippocampus data show a 13% increase from 45% in NDC to 58% AD in the relative abundance of the unedited form INI (Ile157–Asn–159–Ile161). This rise is accompanied by a 12.5% decrease in the relative abundance of the edited form VDV detected in AD. (*B*) Data from temporal lobe of AD (green bars) and NDC (blue bars) show no significant changes in their relative abundance between AD and NDC. (*C*) Data from frontal lobe of AD (green bars) and NDC (blue bars) show no significant changes in their relative abundance between AD and NDC. (Values are represented as mean ± SD. [*] *P* < 0.003, two-way ANOVA).

Several genes within our target-set harbor a number of editing sites located in close proximity. In these cases, the calculated percentage of A/G does not fully reveal the actual impact editing may have on the resulting protein, because it does not provide information on all the possible combinations of editing events. The most prominent example for this phenomenon is the serotonin receptor HTR2C, a member of the large family of seven transmembrane receptors. It harbors five editing sites known as A, B, C′ (previously called E), C and D all residing within 14 bp span of exon 5, corresponding to the second intracellular loop, a region important for G-protein coupling (Burns et al. 1997; Wang et al. 2000; Kawahara et al. 2008). These sites are predicted to occur within amino acid positions 157, 159 and 161. The recoding of these DNA nucleotides by A-to-I RNA editing at these sites, generates up to 32 different mRNA variants that encode for up to 24 different protein isoforms, with varying biochemical properties. The fully edited receptor isoform (corresponding to the amino acids VGV) was shown to have a large reduction of agonist-stimulated G-protein coupling compared with the unedited receptor isoform (corresponding to the amino acids INI) (Berg et al. 2001). The RNA editing levels recorded for each of the four prominent sites A, B, C and D separately did not exhibit a statistically significant reduction in each of the three brain regions studied. However, we noticed that all sites were less edited in AD (Table 4; Fig. 2), suggesting a possible synergetic effect on the distribution of protein variants. Therefore, we analyzed the abundance of each of the 32 transcript variants, and found a statistically significant increase in the level of the un-edited form encoding for INI isoform in AD samples compare with NDC in the HpCpCH. This was accompanied by a 13% decrease in the level of the fully edited VGV version and the partially edited VDV version (Table 5; Fig. 3A–C). These results highlight the importance of studying correlations between neighboring recoding sites, which were by and large overlooked in the past (Barak et al. 2009; Morabito et al. 2010).

**TABLE 4.**
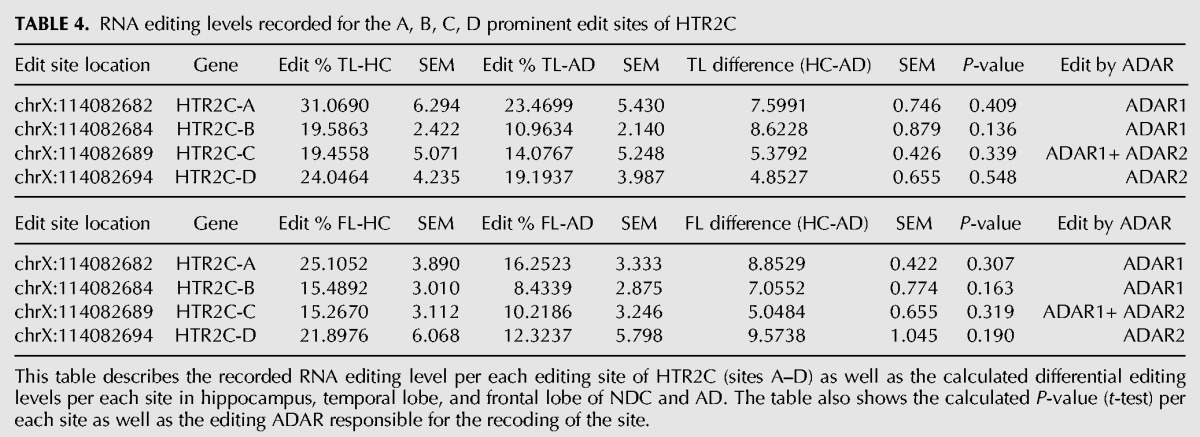
RNA editing levels recorded for the A, B, C, D prominent edit sites of HTR2C

**TABLE 5.**
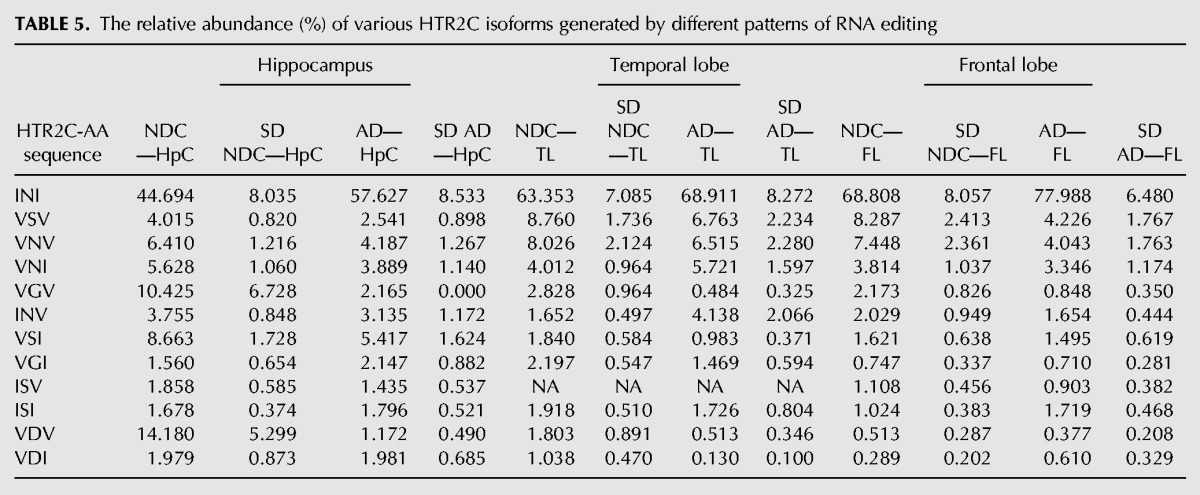
The relative abundance (%) of various HTR2C isoforms generated by different patterns of RNA editing

### Relative mRNA expression of ADAR1 and ADARB1 show a complex pattern of regulation in NDC and AD samples

**FIGURE 4.**
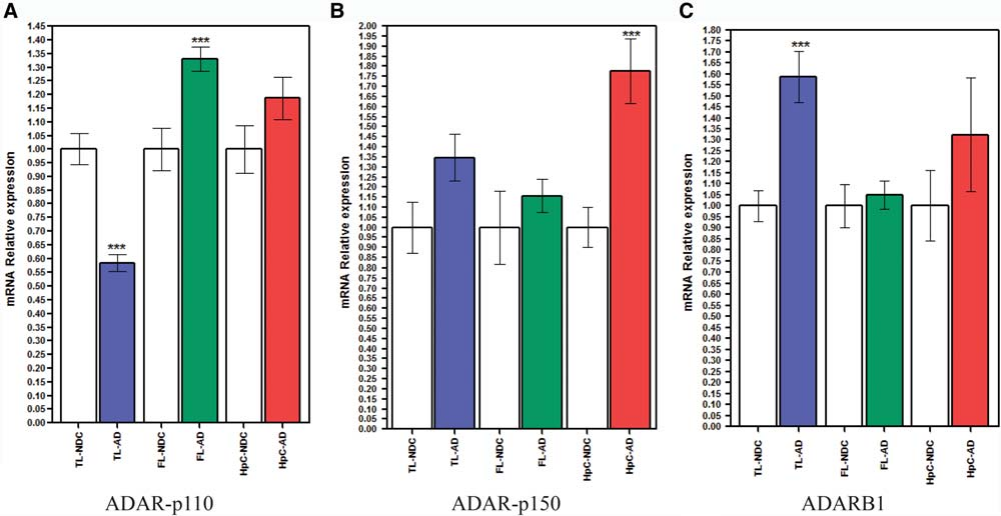
mRNA expression of hADAR1 and hADARB1 in hippocampus, temporal lobe, and frontal lobe of AD. Relative mRNA expression of the two human ADAR1 isoforms, hADAR1-p110 (*A*) and hADAR1-p150 (*B*), and of hADARB1 (*C*) was calculated for hippocampus (red bars), temporal lobe (blue bars), and frontal lobe (green bars) of AD and NDC (blank bars). (*A*) Transcription levels of endogenous hADAR1-p110 are down-regulated in the TL of AD (blue bars) and overexpressed in the FL of AD (green bars). No significant change was detected in the hippocampus. (*B*) Transcription levels of endogenous hADAR-p150 display an overexpression in hippocampus of AD (red bars) compared with their matched NDC (blank bars), while no significant change was detected in the TL or the FL. (*C*) Transcription levels of endogenous hADARB1 show an overexpression in TL of AD (blue bars) compared with their matched NDC (blank bars), while no significant change was detected in the FL or the HpC. Values are represented as means ± SEM. ([***] *P* < 0.001, MW test. *n* = 10 in each group).

Real-time quantitative RT-PCR was performed to calculate the normalized differences in ADAR mRNA levels between hippocampus, frontal and temporal lobes samples of AD to controls. Results are shown in Figure 4A–C. Results show a significant overexpression of hADAR-p150 endogenous transcripts levels in hippocampus (HpC) of AD only, which to some extent is indicative of the neuro-inflammatory processes typical to AD (Fig. 4B). The hADARB1 exhibits an overexpression in AD compare with NDC in the temporal lobe only (Fig. 4C). Lastly, hADAR-p110 isoform shows down-regulation of endogenous transcription levels in AD compare with NDC in the temporal lobe, while displaying an opposite trend of overexpression in the frontal lobe (Fig. 4A). We note that although the global editing levels (mainly reflecting *Alu* editing activity) correlate well with ADAR expression levels, recoding levels present a more complex picture, with no simple correlation to ADAR levels (Maas et al. 2001; Jacobs et al. 2009; Wahlstedt et al. 2009; Garncarz et al. 2013).

## DISCUSSION

Recent massive expansion of transcriptome-wide data allowed for uncovering millions of novel A-to-I RNA editing sites, the vast majority of these reside within repeated sequences and introns (Park et al. 2012; Peng et al. 2012; Ramaswami et al. 2012, 2013; Bazak et al. 2014a). Yet, recoding sites continue to be the focus of most interest, especially as functional impact of RNA editing is discussed. While RNA-seq data present many opportunities in terms of editing detection, it performs rather poorly in quantification, as reads coverage is still not high enough for accurate measurement of the editing level. In cases (such as the one presented here) where the difference between healthy and diseased samples is rather small in terms of editing level, one must resort to alternative methods. Moreover, RNA-seq of hundreds of samples is still rather expensive, and the financial limitation hinders the study of many important conditions. We used an innovative targeted resequencing approach, developed to address these issues, which combines the robustness of microfluidic PCR with deep sequencing output. It enables ultra-high coverage and accurate editing level measurement, while keeping the cost per sample rather low. Thus, it allows for accurate determination of editing levels along with the option to screen across tens of samples on a single run (Zhang et al. 2014). Here we present the first example of using this approach to screen for differences in A-to-I RNA editing between healthy and Alzheimer's disease samples. While clearly one cannot conclude that these differences contribute to AD development, these modifications may corroborate better understanding of the molecular mechanisms underlying the disease, and possibly be utilized as diagnostic markers. We found an overall down-regulation of RNA editing levels in Alzheimer's disease (AD) samples compared with sex- and age-matched healthy nondemented controls (NDC), in all three brain regions screened, and observed site-specific differential editing in 35 sites that are located in 22 different genes. Interestingly, only three sites were found to have hypo-editing levels in AD that is shared by all three regions; the evolutionarily conserved edit site located in the UNC80 gene and the two adjacent sites located in the RNA gene MEG3. Notably, UNC80 is a component of the NALCN sodium channel complex, which regulates its ion conduction in both mammals and invertebrates. Animal models revealed an involvement in many processes such as locomotor behaviors, with mice lacking NALCN exhibited neonatal lethality due to respiratory rhythm defects (Lu et al. 2007; Cochet-Bissuel et al. 2014). Furthermore, hypo-editing in AD was also seen in the noncoding RNA gene LINC-PINT (FLJ43663), which is crucial for proper brain development (Nie et al. 2012; Sauvageau et al. 2013; Guffanti et al. 2014). A strong decrease in editing was observed in the hippocampus (all three sites) and in the temporal lobe (two sites) but not in the frontal lobe. Finally, other eight editing sites also show hypo-editing in AD that is shared by two of the three brain regions tested (Table 6).

**TABLE 6.**
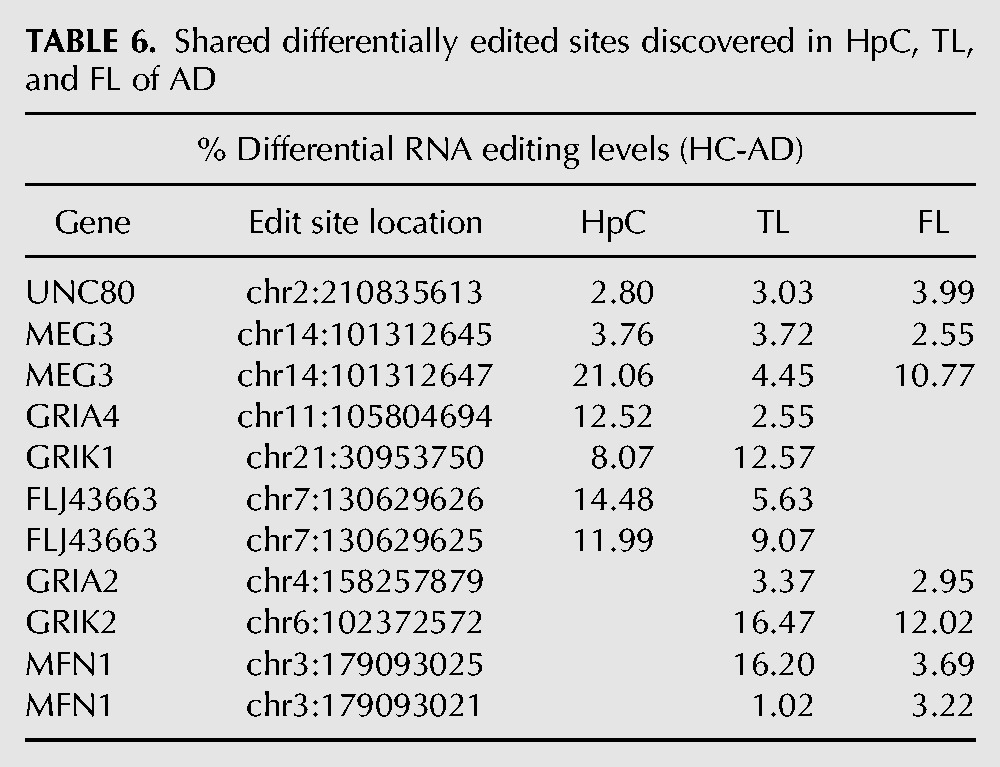
Shared differentially edited sites discovered in HpC, TL, and FL of AD

We found down-regulation of RNA editing in several glutamate receptors: GRIA2 (in TL and FL), GRIA4 (in TL and HpC), and GRIA3 (in HpC). It was already noticed that even mild deregulation of editing levels in these genes may contribute to the demising phenotype of AD (Gaisler-Salomon et al. 2014). The global hypo-editing pattern observed does not fully correlate with ADAR expression patterns, suggesting that more factors are involved. One should also bear in mind that due to motor neuron death, it is possible that the cell-type composition is altered in AD samples, and thus it is possible that while editing level in each cell-type population is not changed in AD, changes in the relative abundance of neurons and glia cells, for example, could have an effect on the global editing level measured. Future studies, using micro-dissection and cell-specific editing quantification, might help to further elucidate this possibility.

## MATERIALS AND METHODS

### Data and classification of brain samples cohorts

Post-mortem samples were collected from three brain regions: hippocampus (HpC), temporal lobe (TL), and frontal lobe (FL) of Alzheimer's disease (AD) and nondemented controls (NDC). The samples were provided by the Netherlands Brain Bank (NBB), Brain and Tissue Bank for Developmental Disorders (NICHD), London Neurodegenerative Diseases Brain Bank (LNDBB), and Parkinson's UK Brain Bank (PUKBB). The AD and NDC samples are gender and age matched. The AD samples were all tested positive for AD symptoms as denoted by the BRAAK score (Table 7; Supplemental Table S3; Braak and Braak 1991).

**TABLE 7.**
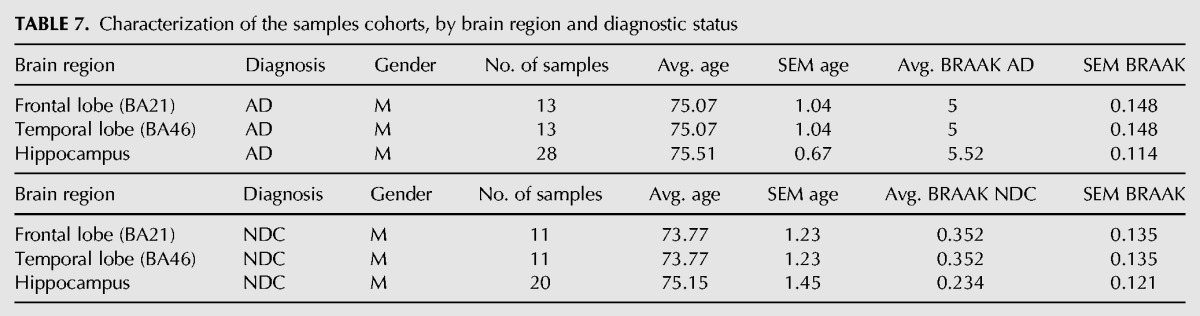
Characterization of the samples cohorts, by brain region and diagnostic status

### RNA and cDNA preparation

Total RNAs were extracted from frozen tissue samples of AD patients and gender- and age-matched nondemented controls obtained from different bio-banks (Supplemental Table S2) using the mirVana miRNA Isolation Kit (Ambion, Life Technologies), according to the procedure supplied by the manufacturers. Total RNA quality was analyzed on the BioAnalyzer 2100 (Agilent), obtaining RIN (RNA Integrity Number) values ranging from 5 to 8, considered acceptable for RNA derived from post-mortem tissues. Six µg of total RNA were retro transcribed using the iScript Advanced cDNA Synthesis Kit for RT-qPCR (Bio-Rad Laboratories Ltd.). The cDNA was purified with MinElute PCR Purification Kit (Qiagen).

### Targeted resequencing of RNA editing sites in RNA samples using the Fluidigm Access Array coupled with the Ion-Torrent PGM

To precisely detect and measure the levels of A-to-I RNA editing in healthy and diseased samples, targeted amplicons were generated and barcoded using a two-step PCR strategy which also minimized the total number of primers required. The target gene specific primers were designed using Primer 3.0 (http://frodo.wi.mit.edu/) to be located in exons while spanning introns, thus avoiding DNA contaminations to the RNA and by the 454 tool for designing of fusion primers supplemented with universal consensus sequences: [http://eu.idtdna.com/scitools/applications/fusionprimers/default.aspx.(IDT).] The primers were tested for specificity and sensitivity by PCR, before they were included in the primers set.

The Fluidigm Access Array is a high-throughput target-enrichment system designed to produce PCR products that could be compatible with all of the major next-generation sequencers. It enables us to create enriched multiple PCR products from 48 samples, all at once. Using the Access Array IFC, we can automatically assemble as little as 2304 PCR reactions, each reaction combining cDNA from one of the 48 samples and one of the 48 primer pairs. The FLDGM-AA amplification and tagging strategy is based on two consecutive PCR reactions, each done with specific fusion PCR primers. The first PCR is performed “on chip” and generates amplicons containing the editing target sites flanked by common universal sequences [CS1 (fused to the forward primer)/CS2 (fused to the reverse primer)]. The second PCR is performed “off chip” using a standard thermal cycler and make use of the first PCR's products as templates. The CS regions previously conjoined (by the previous PCR) enables the attachment of various barcodes to the amplicons, generating longer products. These longer amplicons contains not only the 10 bps sample specific barcode sequences, as well as the Ion-Torrent PGM tr-P1 & A-seq adaptors. Thus, the final PCR output per each sample is a mini-library that is consisted of multiple sequences representing the 48 different target-specific primers pairs, whereas all of them are tagged with the same barcode sequence, which is representative of a single RNA sample. Accordingly, the unified library that is loaded for sequencing is comprised of all 48 mini-libraries represents the entire samples panel.

A schematic representation of the three major steps in the quantification of multiple RNA editing sites by next-generation sequencing (Supplemental Fig. S1): (Step 1) A microfluidics-based PCR using FAA platform generates targeted amplicons from up to 48 samples. Fluidigm Access Array IFC (chip) with samples and primers inlets marked by black arrows. Schematic representation of the “on-chip” PCR; target region (blue lines) that contain targeted RNA editing site (red circle) being amplified by PCR with forward and reverse target-specific primers (TSP-F/TSP-R) fused to common sequences (CS1/CS2). (Step 2) “Off chip” PCR that generates mini-library indexing tagging and the attachment addition of IT-adaptor sequences to create fully tagged and sequencer compatible 48 mini-libraries. Completed amplicons (blue lines flanked by red lines) generated by “off chip” PCR using fusion primers containing CS1 and CS2 (red line of primers) and the Ion-Torrent PGM adaptor sequences P1 (green) and Aseq (orange). Barcode sequences (yellow) for sample indexing are fused to the Aseq-CS2 primer. (Step 3) Parallel sequencing of the combined library on Ion-Torrent PGM using the 1G-318 chip. All 48 mini-libraries representing all 48 samples are constructed of full-length amplicons containing the targeted edit site, barcode sequence for sample identification and sequencer compatible adaptors are pooled together and analyzed on the Ion-Torrent PGM machine.

### Amplification of the target regions containing the target editing sites using the Fluidigm Access Array microfluidic system

Four microliters of single primers pair (4 μM per primer in 1× AA-loading buffer) were loaded into the primer inlets of the 48.48 Access Array IFC (Fluidigm). To prepare the cDNA templates, we added 2.25 μL of each cDNA sample to 2.75 μL of presample mix containing the following enzyme and reagents from the Roche FastStart High Fidelity PCR System; 0.5 μL of 10× FastStart High Fidelity Reaction Buffer wo/Mg, 0.5 μL DMSO (5%), 0.1 μL 10 mM PCR Grade Nucleotide Mix (200 μM), 0.9 μL 25 mM MgCl2 (4.5 Mm), 0.25 μL 20× Access Array Loading Reagent (Fluidigm), 0.05 μL of FastStart High Fidelity Enzyme Blend and 0.7 μL of PCR grade water. Four microliters of this mix was loaded into the samples inlets of the 48.48 Access Array IFC (Fluidigm). After the loading of both samples and primers via IFC Controller AX (Fluidigm) loading script, the IFC was subject to thermal cycling using FC1 Cycler (Fluidigm) with the following program for 40 cycles: 50°C for 2 min, 70°C for 20 min, 95°C 10 min; 10 cycles of: 95°C for 15 sec, 59.5°C for 30 sec, 72°C for 1 min; 4 cycles of: 95°C for 15 sec, 80°C for 30 sec, 59.5°C for 30 sec, 72°C for 1 min; 10 cycles of: 95°C for 15 sec, 59.5°C for 30 sec, 72°C for 1 min; 4 cycles of: 95°C for 15 sec, 80°C for 30 sec, 60°C for 30 sec, 72°C for 1 min; 8 cycles of: 95°C for 15 sec, 59.5°C for 30 sec, 72°C for 1 min; 4 cycles of: 95°C for 15 sec, 80°C for 30 sec, 60°C for 30 sec; 72°C for 1 min; finalizing with 72°C for 3 min. Once PCR has terminated, the IFC was transferred to another IFC Controller AX (Fluidigm) and mini-libraries were harvested by the controller harvest script.

### Sequencing adaptor and barcode addition

For each sample, 1.0 μL of the PCR products harvested from the IFC was 1:110 diluted and added to 15 μL of presample mix containing the following enzyme and reagents from the Roche FastStart High Fidelity PCR System; 2 μL of 10× FastStart High Fidelity Reaction Buffer wo/Mg, 1 μL DMSO (5%), 0.4 μL 10 mM PCR Grade Nucleotide Mix (200 μM), 3.6 μL 25 mM MgCl_2_ (4.5 mM), 0.2 μL of FastStart High Fidelity Enzyme Blend and 7.8 μL of PCR grade water. To that samples mix, 4 μL of primer mix from the 2 μM Access Array Barcode Library for Ion-Torrent PGM Sequencer—96 (P/N100-4911), utilizing the B-set; A–BC–CS2 and P1–CS1 barcode primer combination. We used the following PCR program: 95°C for 10 min; 10 cycles of 95°C for 30 sec, 60°C for 30 sec, and 72°C for 1 min; and 72°C for 5 min.

### Fluidigm library sequencing

Libraries were pooled and sequenced on Ion-Torrent PGM using the Ion PGM Sequencing 200 Kit v2 and the 1G-Ion 318 Chip Kit v2 according to all manufacturer instructions (Life Technologies).

### Bioinformatic sequence analysis

We used the UCSC genome browser Human Feb. 2009 (GRCh37/hg19) assembly for identifying any discrepancies between the Refseq data to that obtained from the actual DNA sequencing output. For our focused screen, we have used a targeted-resequencing approach of NGS (next-generation sequencing) to generate and sequenced multiple PCR amplicons containing the target editing site/s. The analysis of data obtained was performed to detect any A/G mismatches within the cDNA sequences. Such mismatches were summed and scored for their signal strength according to the overall number of coverage reads and more important to the percentage of A-to-G levels.

### Pre-alignment processing

The sequencing data were downloaded from the machine as fastq file. First, all raw sequences data were de-indexed into 48 samples according to the barcodes used by an in-house script. All reads were trimmed of the universal CS1 and CS2 sequences and all short reads (<20 nts) were removed. Alignment of the processed reads was made using bwa version 0.7.4-r385, using the mem option and the parameters: -k 20 -B 3 -O 3 -T 20, for seed in the length of the average primer, and for considering the Ion typical error of small indels.

### Alignments process

The alignment was done to the human refseq database, where reads that were aligned to more than one location were omitted from further analysis. We used samtools mpileup on the alignment results and run in-house script to move the results to the genomic locations from the refseqs and then an in-house script to count the number of different nucleotides in each genomic location that had a *q*-score ≥20. The last stage was to filter the results to a preset set of locations of interest, for each location we present the total number of reads which had good quality per each sample, and the calculated percentage of reads that have a “G” at the specified genomic location, was done accordingly with the formula; (# of “G” reads / [# of “G” reads + # of “A” reads]).

### Real-time PCR quantification assays

Relative mRNA quantification of ADAR-p110 and -p150 variants in addition to ADARB1 was determined using qRT-PCR. Total RNA was extracted and retro transcribed as detailed. For each tested variant, a total of nine biological samples were used. Relative transcript levels were determined by the 7900HT Fast Real-Time PCR System (Applied Biosystems). Triplicates of each cDNA sample were PCR-amplified using the PerfeCTa SYBR Green FastMix (Quanta BioSciences) and the following specific primers:

ADAR-p110: 5′-GGCAGCCTCCGGGTG-3′ and 5′-CTGTCTGTGCTCATAGCCTTG-3′
ADAR-p150: 5′-CGGGCAATGCCTCGC-3′ and 5′-AATGGATGGGTGTAGTATCCGC-3′
ADARB1: 5′-CCGCAGGTTTTAGCTGACG-3′ and 5′-CGGTCAGGTCACCAAACTTACC-3′

The relative quantification of gene expression levels, was measured by the ΔΔC_T_ method (Schmittgen and Livak 2008) and normalized against the SDHA gene (NM_004168.3) using the following gene specific primers; 5′-TTTGATGCAGTGGTGGTAGG-3′ and 5′-TCACGGTGTCGTAGAAATGC-3′. The selection of SDHA for normalization was based on several studies in which it was repeatedly found to be a stable housekeeping gene across various studies, among which included studies that involved AD samples (Eisenberg and Levanon 2013; Jacob et al. 2013; Leidinger et al. 2013; Park et al. 2013). In quantitative real-time PCR assay, gene levels normalizations factors were evaluated by dividing the absolute levels of SDHA gene in each sample by the average value over all NDC samples.

### Statistical analysis

Two-way analysis of variance (ANOVA) was used to compare the expression levels in the AD samples. Editing levels were roughly normally distributed, and no normalization was applied. A *t*-test was used to compare, for each RNA editing site, between editing levels recorded in AD brain samples and brains of nondemented, age and gender matched, healthy controls (NDC). Benjamini–Hochberg multiple testing correction was used with FDR = 0.1. Throughout the paper, values are usually presented as means ± standard error of the mean (SEM).

## SUPPLEMENTAL MATERIAL

Supplemental material is available for this article.

## Supplementary Material

Supplemental Material
