# Supplementary material for: Reduced levels of protein recoding by A-to-I RNA editing in Alzheimer's disease
Source: RNA. 2016 Feb;22(2):290–302. doi: 10.1261/rna.054627.115 (PMC4712678; doi:10.1261/rna.054627.115)
Supplement: Supplemental Material [file supp_22_2_290__index.html]

Reduced levels of protein recoding by A-to-I RNA editing in Alzheimer's disease — Reduced levels of protein recoding by A-to-I RNA editing in Alzheimer's disease — Supplemental Material 

# Reduced levels of protein recoding by A-to-I RNA editing in Alzheimer's disease

## Supplemental Material

**Files in this Data Supplement:**

- Supp Fig S1.pdf
- Supp Table S4.xlsx
- Supp Table S3.xlsx
- Supp Table S1.xlsx
- Supp Fig S1 Legend.docx
- Supp Table S2.xlsx
