# Supplementary figures and images for: Reduced levels of protein recoding by A-to-I RNA editing in Alzheimer's disease
Source: RNA. 2016 Feb;22(2):290–302. doi: 10.1261/rna.054627.115 (PMC4712678; doi:10.1261/rna.054627.115)

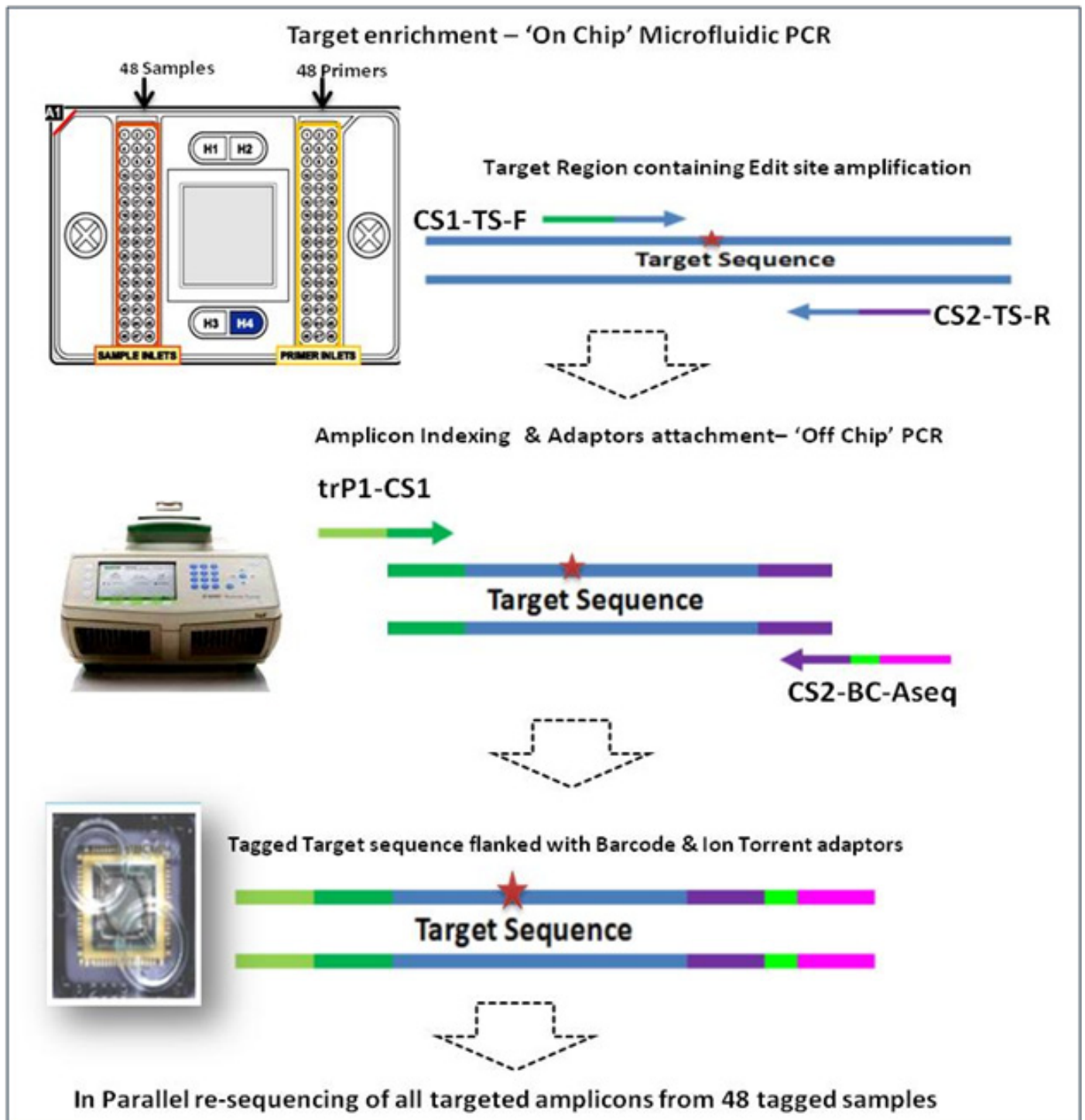

Figure S1

Supplement: Supplemental Material [file supp_054627.115_FigS1.pdf]
