# Supplementary material for: Reduced levels of protein recoding by A-to-I RNA editing in Alzheimer's disease
Source: RNA. 2016 Feb;22(2):290–302. doi: 10.1261/rna.054627.115 (PMC4712678; doi:10.1261/rna.054627.115)
Supplement: Supplemental Material [file supp_054627.115_FigS1_Legend.docx]

Figure S1. Libraries preparation workflow for detecting and quantifying of RNA editing levels of selected target-sites.

Schematic representation of the three major steps in the work process for detection and quantification of multiple RNA editing sites by targeted amplification and next generation sequencing. Step 1: A microfluidics-based PCR using Fl-AA platform generates targeted amplicons from up to 48 samples. Fluidigm Access Array IFC (chip) with samples and primers inlets marked by black arrows. Schematic representation of the “on-chip” PCR; target region (blue lines) that contain targeted RNA editing site (red star) being amplified by PCR with forward and reverse target-specific primers (TSP-F/TSP-R) fused to common sequences CS1 (green line) and CS2 (purple line). Step 2: “Off-chip” PCR that generates 48 mini-library tagging and the addition of IT-adaptor sequences to create fully tagged and sequencer compatible 48 mini-libraries. Completed amplicons (blue lines flanked by green and purple lines) generated by “off chip” PCR using fusion primers containing CS1 and CS2 (green and purple lines of primers) and the Ion Torrent PGM adaptor sequences trP1 (light green) and Aseq (pink). Barcode sequences (lime green) for sample indexing are fused to the Aseq-CS2 primer. Step 3: Parallel sequencing of the combined library on Ion Torrent-PGM using the 1G-318 chip. All 48 mini-libraries representing all 48 samples are constructed of full length amplicons containing the targeted edit site, barcode sequence for sample identification and sequencer compatible adaptors are pooled together and analyzed on the Ion Torrent PGM machine.
